# Working memory characteristics in T2DM individuals with MMSE-defined cognitive status: a behavioral study

**DOI:** 10.3389/fneur.2026.1751377

**Published:** 2026-03-20

**Authors:** Hongyan Bian, Lizheng Gong, Hanxu Qi, Li Yuan, Xu Gao, Yumeng Chen, Qiyu Hu, Zhen Fan, Yanli Su

**Affiliations:** 1 Yan’an Medical College of Yan’an University, Yan’an, China; 2 Department of Psychology, Xi’an Mental Health Center, Xi’an, China

**Keywords:** cognitive status, n-back, type 2 diabetes, verbal, visuospatial, working memory

## Abstract

**Background:**

Cognitive decline is frequently reported in individuals with type 2 diabetes mellitus (T2DM), and working memory impairment represents a key feature of diabetes-related cognitive changes. Previous studies have primarily compared T2DM patients with healthy controls and have typically examined only a single working memory domain. The present study aimed to classify T2DM patients based on Mini-Mental State Examination (MMSE) scores and to examine verbal and visuospatial working memory performance under different cognitive load conditions in order to characterize the cross-sectional association between working memory and global cognitive status.

**Methods:**

Between November 2023 and June 2024, T2DM patients were recruited from the Department of Endocrinology at the Affiliated Hospital of Yan’an University. Based on MMSE scores, participants were classified into a higher cognitive status group (T2DM-HC, *n* = 29, MMSE ≥ 27) and a lower cognitive status group (T2DM-LC, *n* = 25, MMSE 21–26). Working memory performance was assessed using verbal and visuospatial N-back tasks (0-back to 2-back). Reaction time and accuracy were recorded under all task conditions.

**Results:**

Compared with the T2DM-HC group, the T2DM-LC group had a longer duration of diabetes, higher HbA1c levels, and a greater number of comorbidities (all *p* < 0.05). In both verbal and visuospatial N-back tasks, the T2DM-LC group exhibited significantly longer reaction times (all *p* < 0.01), and accuracy declined more markedly under high cognitive load (2-back) conditions (all *p* < 0.001). Partial correlation analyses indicated that MMSE scores were significantly associated only with 2-back accuracy in both verbal and visuospatial conditions (*r* = 0.461, *p* < 0.01; *r* = 0.659, *p* < 0.001). Hierarchical regression analyses showed that inclusion of verbal 2-back accuracy increased the explained variance by 18.9% (Δ*R*^2^ = 0.189), whereas inclusion of visuospatial 2-back accuracy increased the explained variance by 38.6% (Δ*R*^2^ = 0.386).

**Conclusion:**

At the cross-sectional level, verbal and visuospatial working memory performance was significantly associated with global cognitive status in patients with T2DM, with group differences most pronounced under high cognitive load conditions. In this sample, visuospatial 2-back performance demonstrated a numerically stronger association with global cognitive status. This finding is exploratory in nature and warrants further investigation in future studies.

## 1 Introduction

Type 2 diabetes mellitus (T2DM) is a metabolic disease characterized by reduced insulin sensitivity and relative insulin deficiency, accounting for more than 90% of all diabetes cases (1). According to the International Diabetes Federation (IDF) (2), approximately 537 million adults aged 20–79 years were living with diabetes worldwide in 2021, and this number is expected to increase to 783 million by 2045.

Mild cognitive decline, considered a transitional cognitive state between normal aging and dementia, has attracted increasing attention in populations with type 2 diabetes mellitus (T2DM). Diabetes-related cognitive dysfunction, also referred to as diabetes-associated cognitive decline (3), primarily describes cognitive changes observed in individuals with diabetes. Cognitive alterations associated with T2DM may manifest along a continuum ranging from mild cognitive decline to dementia (4). Previous studies have shown that mild cognitive decline occurs at a measurable rate in the general population, and this risk may be further elevated in the context of T2DM (5). Although the prevalence of mild cognitive decline in the general population is approximately 11.9%, it increases to 21.8% among individuals with diabetes (6). Furthermore, an international meta-analysis reported that the prevalence of mild cognitive decline among Asian patients with diabetes reached 46.4%, exceeding that observed in European populations (7). In China, individuals with diabetes are characterized by earlier β-cell dysfunction and a younger age at onset, posing greater challenges compared with Western populations (8, 9). Therefore, elucidating the patterns of cognitive changes in patients with T2DM and exploring objective and sensitive behavioral measures at the research level are of considerable importance.

Individuals with diabetes may exhibit mild cognitive decline, particularly in tasks requiring temporary information maintenance and simultaneous processing (10). Working memory is a core cognitive system supporting such complex cognitive activities and consists of relatively independent verbal and visuospatial subsystems (11). Impairment in working memory has been identified as a prominent feature of diabetes-related cognitive decline, manifested as difficulties in short-term information storage and manipulation, which may subsequently lead to slowed information processing and impaired executive function (12). These deficits not only affect daily functioning but may also complicate glycemic management and increase the risk of falls in individuals with T2DM (13). Neuroimaging studies further demonstrate significant structural atrophy and functional abnormalities in brain regions closely associated with working memory, including the prefrontal cortex, hippocampus, and amygdala. More widespread disruptions in higher-order cognitive networks have been observed in individuals with T2DM who exhibit cognitive decline (14). Collectively, these findings suggest that working memory dysfunction occupies a central position in T2DM-related cognitive changes.

The N-back task is a commonly used paradigm for assessing working memory (15). It requires participants to determine whether the current stimulus matches one of the stimuli presented N items previously, thereby providing a sensitive measure of working memory across different cognitive load levels. Previous studies utilizing the N-back task have suggested that individuals with T2DM may experience working memory impairments; however, the findings have been inconsistent. Regarding verbal working memory, Chen et al. (16) reported no significant differences in accuracy between individuals with T2DM and healthy controls on the 0-back, 1-back, and 2-back digit tasks. In contrast, Cansino et al. (17) using a letter-based N-back task, found higher accuracy rates for T2DM patients under the 1-back and 2-back conditions, with no group differences observed under the 0-back condition. Similar inconsistencies have also been reported in visuospatial working memory research (18, 19). These discrepancies may reflect variations in participant characteristics (such as age and baseline cognitive ability), as well as differences in experimental materials (e.g., digits, letters, or shapes) and task parameters (including stimulus duration and interstimulus intervals). This methodological heterogeneity complicates direct comparisons across studies, making it unclear under which cognitive load conditions differences in N-back performance are most pronounced within T2DM populations with varying cognitive status.

Moreover, verbal and visuospatial working memory rely on partially distinct neural mechanisms. Verbal working memory predominantly involves left-hemispheric networks and maintains information through rehearsal processes that actively refresh temporary representations, thereby preventing decay (20). In contrast, visuospatial working memory depends more heavily on right-hemispheric networks and is primarily maintained through selective attention mechanisms that require inhibition of task-irrelevant information. When attentional resources are limited and competition for cognitive resources occurs, visuospatial working memory may be particularly vulnerable (21). Given that individuals with T2DM who exhibit cognitive decline may have constrained attentional resources and vulnerable neural networks, performance across different types of working memory tasks may not be uniform and warrants further empirical investigation.

Although research in this area has gradually shifted from simple cross-sectional comparisons with healthy controls to a greater focus on within-group differences among individuals with T2DM (e.g., varying HbA1c levels) (22), studies examining T2DM populations stratified by cognitive functioning remain limited. In addition, most existing research has examined verbal and visuospatial working memory separately, with relatively few studies integrating both domains within a single experimental framework.

To address these gaps, the present study employed the N-back paradigm to systematically examine verbal and visuospatial working memory performance under different levels of cognitive load in T2DM participants categorized into a lower cognitive status group (T2DM-LC) and a higher cognitive status group (T2DM-HC) based on MMSE scores. Furthermore, we analyzed the relationship between N-back performance and MMSE scores to explore, from a cross-sectional behavioral perspective, the correspondence between objective working memory task performance and global cognitive functioning.

This study primarily aimed to address two questions: (1) Under which cognitive load conditions do performance differences between the T2DM-LC and T2DM-HC groups emerge in the N-back task? and (2) Are accuracy rates in verbal and visuospatial N-back tasks significantly associated with MMSE scores? We hypothesized that the T2DM-LC group would demonstrate poorer performance under higher cognitive load conditions and that N-back accuracy would be significantly correlated with MMSE scores. If supported, these findings would help characterize working memory performance patterns across different levels of cognitive functioning in T2DM and provide a basis for further investigation into the relationship between objective cognitive task performance and global cognitive status indicators.

## 2 Methods

### 2.1 Participants

From November 2023 to June 2024, patients with type 2 diabetes mellitus (T2DM) were recruited from the inpatient ward of the Department of Endocrinology at the Affiliated Hospital of Yan’an University. The required sample size was estimated using G*Power 3.1.9.2 (Cohen’s *f* = 0.25, *α* = 0.05, power = 0.95). For the 2 (T2DM-HC vs. T2DM-LC) × 3 (0-back, 1-back, 2-back) mixed design analysis, the minimum required sample size was calculated to be 44 participants. A total of 60 patients were recruited for the study. Among them, 4 were excluded due to insufficient education levels, which prevented them from completing the cognitive tasks; 1 patient was excluded due to a history of stroke; and 1 participant withdrew during the course of the study. Ultimately, 54 patients completed the entire study protocol and were included in the final analysis. This study was approved by the Ethics Committee of the Affiliated Hospital of Yan’an University (approval number: YA-L20240004) and was conducted in accordance with the Declaration of Helsinki. Prior to participation, the study procedures and objectives were explained to all participants and their families, and written informed consent was obtained after confirming their understanding. For participants with lower MMSE scores or suspected cognitive difficulties, additional consent was obtained from their families.

### 2.2 Inclusion and exclusion criteria

Inclusion criteria were as follows: (1) age ≥ 18 years; (2)sufficient educational level and comprehension ability to complete the cognitive task(primary school education or above); (3) a diagnosis of type 2 diabetes mellitus in accordance with the 2020 Chinese Guidelines for the Prevention and Treatment of Type 2 Diabetes, defined by the presence of any one of the following criteria: (a) typical diabetic symptoms with a random plasma glucose level ≥ 11.1 mmol/L; (b) fasting plasma glucose ≥ 7.0 mmol/L; or (c) a 2-h plasma glucose level ≥ 11.1 mmol/L during an oral glucose tolerance test (OGTT); and (4) provision of written informed consent by the participant and their family members, with the ability to complete all study procedures.

Exclusion criteria included: (1) significant visual, auditory, speech, or motor impairments that could interfere with task performance or button responses; (2) a documented history of central nervous system disorders, such as stroke, traumatic brain injury, or neurodegenerative diseases; (3) a history of severe psychiatric disorders, including major depressive disorder, bipolar disorder, or schizophrenia; (4) use of medications known to affect cognitive function within a recent period prior to assessment (e.g., within 4 weeks); (5) presence of acute diabetic complications, such as diabetic ketoacidosis or hyperosmolar hyperglycemic state; (6) comorbid malignant tumors; (7) significant dysfunction of major organs, including the heart, liver, or kidneys; (8) a history of alcohol dependence; and (9) occurrence of hypoglycemic episodes, severe hyperglycemia, or active infection within 24 h prior to cognitive assessment.

To minimize the potential impact of neurological or psychiatric conditions on cognitive performance, participants were screened using a multi-step procedure. First, two trained researchers independently reviewed complete inpatient electronic medical records to confirm the absence of relevant neurological or psychiatric diagnoses, hospitalization records, or medication histories. Second, structured interviews were conducted by trained researchers in accordance with the Diagnostic and Statistical Manual of Mental Disorders, Fifth Edition (DSM-5) to screen for psychiatric disorders. Participants with clinically significant depressive symptoms or those currently receiving antidepressant treatment were excluded. Depressive symptom severity was not quantitatively assessed in the present study, as depression was considered solely as an exclusion criterion rather than as a continuous variable in the statistical analyses.

### 2.3 Grouping criteria

The MMSE was used to assess overall cognitive function and served as the basis for cognitive status grouping. Developed by Folstein et al. (23), the MMSE consists of 30 items, covering five domains: orientation, attention and calculation, memory, recall, and language, with a total score range of 0–30; higher scores indicate better overall cognitive function. Previous studies have shown that the MMSE has good internal consistency (Cronbach’s *α* = 0.81) and is widely used for the quantitative assessment of overall cognitive function (24).

In this study, MMSE scores were used solely as an operational criterion for group classification. Participants with MMSE scores of 21–26 were categorized into the T2DM-LC group (*n* = 25), whereas those with MMSE scores ≥27 were categorized into the T2DM-HC group (*n* = 29). It should be emphasized that group classification in this study was based exclusively on MMSE cut-off values for research purposes and does not constitute a formal clinical diagnosis of cognitive impairment according to comprehensive clinical criteria.

Furthermore, the MMSE primarily reflects global cognitive functioning and has limited sensitivity to subtle changes in specific cognitive domains, such as working memory. MMSE total scores may also be influenced by demographic factors, including age, sex, and years of education. Therefore, age, sex, and years of education were included as covariates in subsequent statistical analyses to control for potential confounding effects.

### 2.4 Data collection

#### 2.4.1 Clinical data collection

Data for all participants were collected through the electronic medical record system, including the following:

1. Demographic Information: age, sex, educational level, economic status, marital status, smoking and drinking history.
2. Clinical Characteristics: height, weight, body mass index (BMI), disease duration, systolic blood pressure (SBP), diastolic blood pressure (DBP), fasting plasma glucose (FPG), hemoglobin A1c (HbA1c), number of comorbidities, and treatment regimen (insulin, metformin, insulin combined with metformin).

The glycemic treatment during hospitalization was determined and adjusted by the attending clinicians based on individual circumstances. This study did not intervene in the treatment regimen; relevant medication information was used solely for sample description and, when necessary, included in the analysis as a potential confounding factor.

#### 2.4.2 Working memory assessment

Working memory was assessed using verbal and visuospatial N-back tasks programmed in E-Prime 3.0. The N-back paradigm, originally developed by Kirchner (25), requires participants to determine whether the current stimulus matches the stimulus presented N trials earlier. Stimuli were presented on a 15.3-inch Lenovo ThinkBook laptop computer (resolution: 1024 × 768) with a white background and black text. The viewing distance was fixed at 50 cm. All tasks were administered in a quiet environment, and standardized instructions were provided to all participants.

##### 2.4.2.1 Verbal N-back task

**Figure 1:**
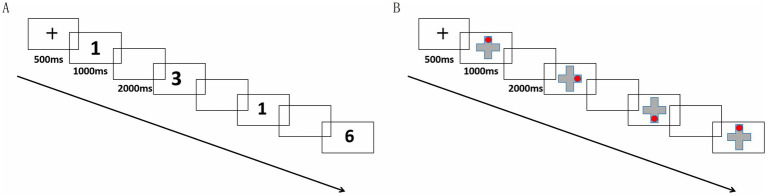
Flowchart of the verbal and visuospatial N-back tasks: **(A)** Flowchart of the verbal N-back task; **(B)** Flowchart of the visuospatial N-back task.

In the verbal N-back task, Arabic numerals were used as stimuli, and participants were required to determine whether the currently presented number matched the number presented N trials earlier. The task included three levels of cognitive load (0-back, 1-back, and 2-back). Following previous research (16), each trial began with a 500 ms fixation point, followed by a 1,000 ms stimulus presentation, with a 2,000 ms interstimulus interval and a maximum response window of 3,000 ms. The practice phase consisted of 10 trials, while the formal experimental phase included 30 trials, with a target-to-nontarget trial ratio fixed at 1:2. Each cognitive load condition lasted approximately 7 min, with a 1-min rest interval between different load conditions, resulting in a total task duration of about 10 min (Figure 1). The verbal and visuospatial N-back tasks were presented in a fixed order for all participants. Within each task, cognitive load conditions (0-back, 1-back, 2-back) were presented in a fixed sequence from low to high to reduce the initial task load for older participants.

##### 2.4.2.2 Visuospatial N-back task

The visuospatial N-back task used a gray cross and a red circle appearing at pseudorandom locations on the screen. Participants were required to determine whether the location of the current stimulus matched the location presented N trials earlier. Trial timing parameters, number of trials, and rest intervals were identical to those used in the verbal N-back task.

##### 2.4.2.3 Experimental design and data processing

A single-blind design was employed, such that researchers administering the N-back tasks were unaware of participants’ group assignments (T2DM-LC or T2DM-HC). Behavioral data, including accuracy and reaction time, were automatically recorded by E-Prime. Individual trials were excluded according to predefined criteria: accuracy <50%, reaction time <200 ms, or values exceeding ±2.5 standard deviations from the participant’s mean (26, 27), to ensure data quality.

### 2.5 Statistical analysis

Statistical analyses were performed using SPSS version 26.0. All tests were two-tailed, and the significance level was set at *p* < 0.05. The normality of continuous variables was assessed before analysis. Normally distributed variables were expressed as mean ± standard deviation and compared between groups using independent *t*-tests. Non-normally distributed variables were expressed as median (P25, P75) and compared using the Mann–Whitney U test. Categorical variables were presented as counts (percentages) and analyzed using the chi-square test, with Fisher’s exact test used when appropriate.

A mixed-design repeated measures analysis of variance (ANOVA) was conducted, with group (T2DM-LC vs. T2DM-HC) as the between-subjects factor and cognitive load (0-back, 1-back, 2-back) as the within-subjects factor, to examine the main effects and interaction effects of verbal and visuospatial N-back task performance. Greenhouse–Geisser correction was applied when the sphericity assumption was violated. Simple effects analysis was performed only when the interaction was significant, and Bonferroni correction was applied in post-hoc comparisons between groups under different cognitive load conditions. Additionally, partial correlation analysis was conducted to examine the relationship between N-back accuracy and MMSE scores, controlling for age, gender, and years of education. Further, hierarchical regression analysis was performed, with demographic variables entered in the first step and N-back accuracy in the second step, to test the association between working memory task performance and MMSE scores after controlling for demographic factors. The above correlation and regression analyses were exploratory in nature and addressed different research questions, and no global multiple comparison correction was applied, which may increase the risk of Type I error.

## 3 Results

### 3.1 Baseline characteristics of the study population

Among the 54 T2DM participants included in the analysis, 25 were classified into the T2DM-LC group and 29 into the T2DM-HC group based on the MMSE operational grouping criteria. Compared to the T2DM-HC group, participants in the T2DM-LC group had a longer duration of diabetes (13.0 vs. 10.0 years), higher HbA1c levels (9.0% vs. 8.0%), more comorbidities (2.0 vs. 1.0), and lower MMSE scores (24 vs. 28). All group differences were statistically significant (*p* < 0.05) (Table 1).

**Table 1:** Baseline characteristics of patients with T2DM-LC and T2DM-HC.

Characteristics	T2DM-HC (*n* = 29)	T2DM-LC (*n* = 25)	t/Z/*χ*^2^	*p*
Age (y)	57.72 ± 4.86	58.84 ± 4.90	−0.838^a^	0.406
Gender			1.862^c^	0.172
Male, *n* (%)	17 (58.6)	10 (40.0)		
Female, *n* (%)	12 (41.4)	15 (60.0)		
Duration of education			2.658^b^	0.447
Primary school, *n* (%)	5 (17.2)	7 (28.0)		
Junior high school, *n* (%)	14 (48.3)	14 (56.0)		
High school, *n* (%)	8 (27.6)	3 (12.0)		
College degree or above, *n* (%)	2 (6.9)	1 (4.0)		
Economic situation (yuan/month)			0.739^b^	0.691
<3,000	8 (27.6)	9 (36.0)		
3,000 ~ 5,000	14 (48.3)	12 (48.0)		
>5,000	7 (24.1)	4 (16.0)		
Marital status			1.182^c^	0.463
Married, *n*(%)	29 (100.0)	24 (96.0)		
Widowhood and Other, *n*(%)	0 (0.00)	1 (4.0)		
Treatment regimen			1.293^c^	0.524
Insulin, *n* (%)	12 (41.4)	7 (28.0)		
Metformin, *n* (%)	8 (27.6)	7 (28.0)		
Insulin plus metformin, *n* (%)	9 (31.0)	11 (44.0)		
Smoking, *n* (%)	14 (48.3)	8 (32.0)	2.241^c^	0.524
Drinking, *n* (%)	18 (62.1)	15 (60.0)	1.359^c^	0.715
BMI(kg/m^2^)	23.41 ± 1.92	24.48 ± 2.88	−1.571^a^	0.124
SBP (mmHg)	127.34 ± 14.75	126.92 ± 12.68	0.113^a^	0.911
DBP (mmHg)	79.45 ± 8.26	77.32 ± 7.94	0.962^a^	0.341
Duration of diabetes (y)	10.00 (8.00, 13.00)	13.00 (11.00, 17.00)	−2.525^b^	0.012
FPG (mmol/L)	8.00 (7.00, 9.50)	9.00 (8.00, 10.50)	−1.470^b^	0.142
HbA1c (%)	8.00 (8.00, 9.00)	9.00 (8.00, 10.50)	−2.469^b^	0.014
Number of complications	1.00 (0.00, 2.00)	2.00 (1.00, 2.00)	−2.461^b^	0.017
MMSE	28 (27, 29)	24 (22.5, 24.5)	−6.362^b^	<0.001

Data are presented as mean ± standard deviation (SD), median (interquartile range) [M (P25, P75)], or as number (percentage).

BMI, body mass index; SBP, systolic blood pressure; DBP, diastolic blood pressure; FPG, fasting plasma glucose; HbA1c, glycosylated hemoglobin; MMSE: Mini-Mental State Examination; ^a^Independent samples *t*-test. ^b^Mann–Whitney U test (*Z* value reported). ^c^Chi-square test (*χ*^2^).

During hospitalization, two patients (one from the T2DM-LC group and one from the T2DM-HC group) had received sedative medications, and one patient (from the T2DM-HC group) was admitted through the emergency department. These patients were excluded from the sensitivity analysis.

### 3.2 Descriptive performance on the N-back tasks

In the verbal N-back task, the T2DM-LC group demonstrated longer reaction times than the T2DM-HC group across all cognitive load conditions (0-back: 882.3 vs. 787.6 ms; 1-back: 1179.2 vs. 995.9 ms; 2-back: 1577.0 vs. 1347.9 ms). With respect to accuracy, no clear group differences were observed under the 0-back or 1-back conditions; however, accuracy was lower in the T2DM-LC group under the 2-back condition (64.7% vs. 69.6%) (Table 2).

**Table 2:** Reaction time and accuracy of subjects in verbal and visuospatial N-back tasks.

Load level	T2DM-HC (*n* = 29)	T2DM-LC (*n* = 25)	*t*	MD (95% CI)	Cohen’s d	*p*
Verbal N-back- Reaction time (ms)						
0-back	787.56 ± 118.13	882.32 ± 139.90	−2.70	−94.76 (−165.21, −24.31)	0.74	0.009
1-back	995.89 ± 95.63	1179.15 ± 133.12	−5.87	−183.26 (−245.95, −120.57)	1.60	<0.001
2-back	1347.94 ± 109.04	1577.02 ± 116.15	−7.47	−229.09 (−290.63, −167.54)	2.04	<0.001
Verbal N-back- Accuracy (%)						
0-back	96.55 ± 3.19	95.58 ± 3.52	1.06	0.97 (−0.87, 2.80)	0.29	0.296
1-back	88.47 ± 6.47	86.26 ± 5.30	1.35	2.20 (−1.06, 5.47)	0.37	0.182
2-back	69.60 ± 5.11	64.69 ± 4.37	3.76	4.91 (2.29, 7.529)	1.03	<0.001
Visuospatial N-back-Reaction time (ms)						
0-back	850.35 ± 123.96	957.92 ± 153.13	−2.85	−107.57 (−183.25, −31.89)	0.78	0.006
1-back	1109.18 ± 133.47	1347.25 ± 118.38	−6.88	−238.07 (−307.48, −168.67)	1.88	<0.001
2-back	1446.47 ± 121.54	1663.55 ± 118.43	−6.62	−217.08 (−282.86, −151.30)	1.81	<0.001
Visuospatial N-back- Accuracy (%)						
0-back	95.71 ± 3.28	94.07 ± 4.28	1.60	1.65 (−0.42, 3.72)	0.43	0.115
1-back	87.24 ± 6.11	84.66 ± 5.82	1.58	2.58 (−0.69, 5.86)	0.43	0.120
2-back	65.75 ± 4.11	58.39 ± 4.61	6.21	7.37 (4.99, 9.75)	1.69	<0.001

Data are mean ± SD unless otherwise indicated; MD, mean difference; CI, confidence interval; Cohen’s *d* represents the standardized effect size for between-group comparisons.

In the visuospatial N-back task, a comparable pattern was observed. The T2DM-LC group showed longer reaction times across all load levels, and differences in accuracy were primarily observed under the 2-back condition (Table 2).

### 3.3 Verbal N-back task performance in patients with T2DM

Based on the previous analysis, the verbal and visuospatial N-back task performances of the T2DM-LC and T2DM-HC groups were further compared.

**Figure 2:**
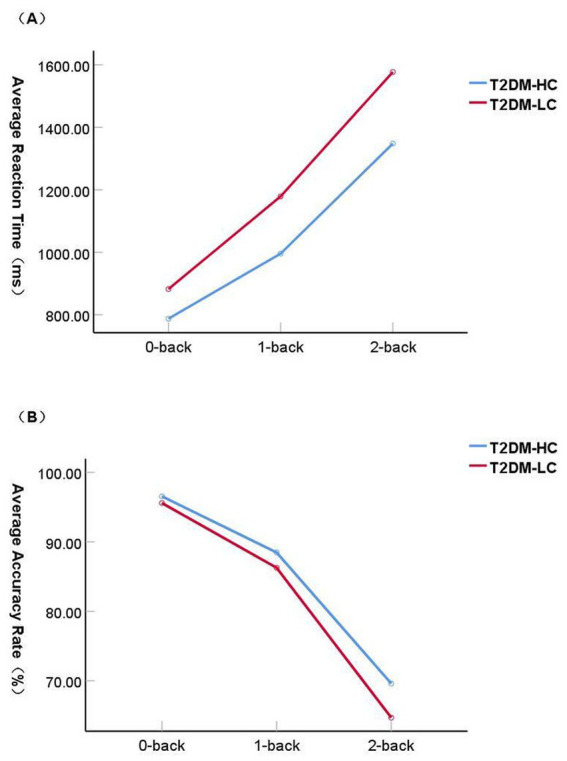
Verbal N-back task performance in the T2DM-HC and T2DM-LC groups. **(A)** Average reaction time. **(B)** Average accuracy. Data are from cross-sectional group comparisons based on a single assessment. The line plots are for illustrative purposes, displaying descriptive patterns of performance under different task loads, and do not represent longitudinal changes or causal effects.

Reaction Time: Repeated measures analysis of variance (ANOVA) revealed a significant main effect of group [*F*(1, 52) = 66.926, *p* < 0.001, ηp^2^ = 0.563], a significant main effect of cognitive load [*F*(2, 104) = 426.878, *p* < 0.001, ηp^2^ = 0.891], and a significant group × load interaction [F(2, 104) = 4.991, *p* < 0.01, ηp^2^ = 0.088]. Simple effects analysis indicated that, under the 0-back, 1-back, and 2-back conditions, the reaction time of the T2DM-LC group was significantly longer than that of the T2DM-HC group (all *p* < 0.01). Additionally, reaction times for both groups significantly increased as task load increased (*p* < 0.001) (Table 3; Figure 2).

**Table 3:** Repeated measures ANOVA table for the verbal and visuospatial N-back tasks.

Task	Effect	SS	MS	*F*	*p*	ηp^2^
Verbal N-back reaction time (ms)	Group^a^	1150860.883	1150860.883	66.926	<0.001	0.563
	Load level^b^	10708515.79	5354257.896	426.878	<0.001	0.891
	Group*Load level^c^	125193.855	62596.928	4.991	0.009	0.088
Verbal N-back accuracy (%)	Group^a^	291.908	291.908	9.108	0.004	0.149
	Load level^b^	23648.920	11824.460	629.601	<0.001	0.924
	Group*Load level^c^	525.989	304.388	15.353	<0.001	0.228
Visuospatial N-back reaction time (ms)	Group^a^	1417128.830	1417128.830	60.707	<0.001	0.539
	Load level^b^	11375473.51	5687736.757	433.490	<0.001	0.893
	Group*Load level^c^	131857.506	65928.753	5.025	0.008	0.088
Visuospatial N-back accuracy (%)	Group^a^	602.206	602.206	15.738	<0.001	0.232
	Load level^b^	30924.001	15462.001	1008.357	<0.001	0.951
	Group*Load level^c^	252.589	126.294	8.263	<0.001	0.137

^a^represents the main effect of group. ^b^represents the main effect of load level, and ^c^represents the interaction effect between group and load level; SS, sum of squares; df, degrees of freedom; MS, mean square; ηp^2^, partial eta squared.

Accuracy: For accuracy, repeated measures ANOVA showed a significant main effect of group [*F*(1, 52) = 9.108, *p* < 0.01, ηp^2^ = 0.149], a significant main effect of cognitive load [*F*(2, 104) = 629.601, *p* < 0.001, ηp^2^ = 0.924], and a significant group × load interaction [F(2, 104) = 15.353, *p* < 0.001, ηp^2^ = 0.228]. Simple effects analysis revealed that the T2DM-LC group exhibited significantly lower accuracy than the T2DM-HC group only under the 2-back condition (*p* < 0.001), while accuracy for both groups significantly decreased as cognitive load increased (Table 3; Figure 2).

### 3.4 Visuospatial N-back task in patients with T2DM

Building on the preceding analyses, visuospatial N-back task performance was further compared between the T2DM-LC and T2DM-HC groups.

**Figure 3:**
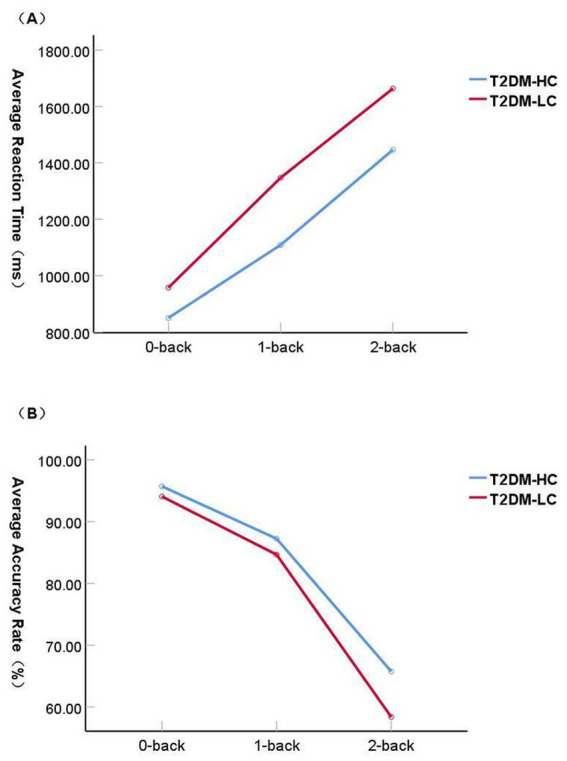
Visuospatial N-back task performance in the T2DM-HC and T2DM-LC groups. **(A)** Average reaction time. **(B)** Average accuracy. Data are from cross-sectional group comparisons at a single time point. The trends shown in the figure reflect only descriptive differences under different load conditions and do not suggest longitudinal progression or causal relationships.

Reaction time: Repeated-measures analysis of variance revealed a significant main effect of group [*F*(1, 52) = 60.707, *p* < 0.001, ηp^2^ = 0.539], a significant main effect of cognitive load [*F*(2, 104) = 433.490, *p* < 0.001, ηp^2^ = 0.893], and a significant group × load interaction [*F*(2, 104) = 5.025, *p* < 0.01, ηp^2^ = 0.088]. Follow-up simple effects analyses with Bonferroni correction (*α*′ = 0.017) indicated that reaction times were longer in the T2DM-LC group than in the T2DM-HC group under the 0-back, 1-back, and 2-back conditions (all *p* < 0.01). In addition, reaction times increased significantly with increasing task load in both groups (*p* < 0.001) (Table 3; Figure 3).

Accuracy: For accuracy, repeated-measures analysis of variance showed significant main effects of group [*F*(1, 52) = 15.738, *p* < 0.001, ηp^2^ = 0.232] and cognitive load [*F*(2, 104) = 1008.357, *p* < 0.001, ηp^2^ = 0.951], as well as a significant group × load interaction [F(2, 104) = 8.263, *p* < 0.001, ηp^2^ = 0.137]. Simple effects analyses indicated that the T2DM-LC group exhibited lower accuracy than the T2DM-HC group only under the 2-back condition (*p* < 0.001), whereas accuracy decreased significantly with increasing cognitive load in both groups (Table 3; Figure 3).

### 3.5 Partial correlations between N-back task accuracy and MMSE scores

To examine the associations between working memory performance and global cognitive function, partial correlation analyses were conducted between N-back task accuracy and MMSE scores after controlling for age, sex, and years of education. The results showed that MMSE scores were significantly positively correlated with accuracy on the verbal 2-back task (*r* = 0.461, *p* < 0.01) and the visuospatial 2-back task (*r* = 0.659, *p* < 0.001). No significant correlations were observed between MMSE scores and task accuracy under the 0-back or 1-back conditions (Table 4).

**Table 4:** Partial correlation analysis results of verbal and visuospatial working memory tasks.

Load level	*r*	*p*
Verbal N-back task accuracy (%)		
0-back	0.135	0.346
1-back	0.146	0.306
2-back	0.461	0.001
Visuospatial N-back task accuracy (%)		
0-back	0.127	0.373
1-back	0.151	0.291
2-back	0.659	<0.001

### 3.6 Hierarchical regression analysis of N-back task accuracy and MMSE scores

To further examine the association between N-back task accuracy and MMSE scores after controlling for demographic factors, hierarchical regression analysis was performed. Prior to model estimation, the basic assumptions of regression analysis were tested, and the results indicated that the data met the primary requirements for linear regression, including linear relationships, residual independence, and the absence of significant multicollinearity.

MMSE scores were used as the dependent variable. In Model 1, age, gender, and years of education were included as covariates in the regression model. In Model 2, after controlling for the aforementioned demographic variables, the accuracy of verbal 2-back and visuospatial 2-back tasks was included separately. The results showed that, after controlling for demographic factors, the inclusion of 2-back task accuracy significantly improved the model’s ability to explain MMSE scores. Specifically, the inclusion of verbal 2-back task accuracy increased the model’s explanatory power by 18.9% (ΔR^2^ = 0.189, *p* < 0.01), while the inclusion of visuospatial 2-back task accuracy increased the model’s explanatory power by 38.6% (ΔR^2^ = 0.386, *p* < 0.001) (Table 5).

**Table 5:** Hierarchical regression analysis results of verbal and visuospatial working memory tasks.

Variable	Verbal task		Visuospatial task	
	Model 1	Model 2	Model 1	Model 2
Constant	1.674**	1.570**	1.674**	1.681**
Age	−0.040	−0.132	−0.040	−0.116
Sex	−1.044	−1.005	−1.044	−1.411
Education	0.539	0.311	0.539	0.072
2-back accuracy	/	0.260**	/	0.316***
*R* ^2^	0.112	0.301**	0.112	0.498***
Δ*R*^2^	0.112	0.189**	0.112	0.386***
*F*	2.112	5.285**	2.112	12.152***

(1) The table shows unstandardized regression coefficients (B). (2) Model 1 includes age, sex, and years of education as control variables. Model 2 further includes 2-back task accuracy. (3) The incremental contribution of working memory performance to the model is assessed through the change in explained variance (Δ*R*^2^), rather than directly comparing the size of the regression coefficients. (4) *p < 0.05, **p < 0.01, ***p < 0.001. (5) “Accuracy” in the table refers to the behavioral performance metric of the 2-back task and is not used as a clinical diagnostic criterion.

To evaluate the potential impact of disease severity on reaction time outcomes, we conducted additional sensitivity analyses. When only disease severity indicators were included in the models, none of these variables were significantly associated with reaction time. However, after further adding 2-back accuracy, it was significantly negatively associated with reaction time in both the verbal and visuospatial tasks (all *p* ≤ 0.003), and the model explanatory power increased substantially (ΔR^2^ = 0.16–0.29) (see Supplementary Table S4).

To further examine the robustness of the findings, we performed parallel sensitivity analyses by separately adding diabetes duration, HbA1c, and number of comorbidities to the base model controlling for age, sex, and years of education. Across all sensitivity models, the group differences in 2-back accuracy remained in the same direction, with only minimal changes in effect size, and statistical significance was consistently preserved (all *p* ≤ 0.003), indicating that the primary findings were not confounded by indicators of disease severity (see Supplementary Table S5).

## 4 Discussion

### 4.1 Working memory characteristics in T2DM individuals with different cognitive status

The present study systematically examined verbal and visuospatial working memory in T2DM-LC individuals using the N-back paradigm. Compared with the T2DM-HC group, T2DM-LC individuals exhibited prolonged reaction times across both task modalities, whereas significant between-group differences in accuracy were observed only under the high-load (2-back) condition. These findings suggest that working memory impairment in T2DM individuals with lower cognitive status is load-dependent and becomes more pronounced as cognitive demands increase. This pattern is consistent with findings reported by Dubois et al. (28), who observed reduced accuracy under high-load verbal working memory conditions in older adults with clinically defined cognitive impairment, suggesting that performance differences between individuals with varying levels of cognitive performance are more likely to emerge under higher task demands.

To further elucidate these findings, we explored potential mechanisms underlying the performance deficits observed under high cognitive load. The significant behavioral differences detected in the 2-back condition may be attributable to the increased demand on limited cognitive resources as task complexity intensifies. Previous research has suggested that, when cognitive demands rise, constraints in the flexible allocation of neural resources within working memory–related brain regions, together with increased interference from task-irrelevant information, may contribute to performance decline (18). It should be noted that the present study was behavioral in nature; therefore, these interpretations remain speculative.

It is noteworthy that no significant between-group differences were observed under the low-load (1-back) condition. This finding may be related to characteristics of the study population. T2DM itself has been associated with nonspecific cognitive decline or subtle cognitive alterations; even individuals who do not meet clinical diagnostic criteria may already exhibit a certain degree of reduced cognitive reserve (29). Under moderate cognitive load, task performance may be maintained through strategic adjustments or the reallocation of cognitive resources. This interpretation is supported by the findings of Fraga et al. (30) and is consistent with cognitive resource theory (31). Alternative explanations should also be considered. For instance, the generalized slowing in reaction time may reflect deficits in sustained attention or vigilance, whereas the selective reduction in accuracy under high-load conditions may be attributable to limitations in working memory capacity or increased susceptibility to interference.

The above findings are indirectly supported by research in other clinical populations. Previous studies have shown that individuals with clinically defined cognitive impairment are more likely to exhibit deficits under high-load working memory conditions. Hattori et al. (32) reported in a functional neuroimaging study that cognitively normal patients with Parkinson’s disease (PD) demonstrated increased activation in working memory-related brain regions under high-load task conditions, whereas PD patients with clinically diagnosed cognitive impairment exhibited a similar activation pattern under lower-load conditions. This pattern has been interpreted as reflecting reduced efficiency in cognitive resource utilization or earlier recruitment of compensatory mechanisms; however, the underlying mechanisms remain under debate.

Although the present study did not involve formal clinical diagnostic classification of cognitive impairment and did not include neuroimaging measures, the performance decline observed under high-load conditions in T2DM individuals with lower cognitive status appears behaviorally similar to the load-sensitive patterns reported in prior studies. This behavioral consistency may suggest that across different disease contexts, individuals with cognitive decline are more likely to exhibit load-dependent alterations in working memory performance under increased task demands.

One possible explanation is that as cognitive demands increase, available cognitive resources more readily approach their capacity limits, resulting in reduced behavioral efficiency under high-load conditions (33). Some neuroimaging studies have proposed that this phenomenon may be related to functional reorganization or compensatory modulation among brain regions involved in working memory (34). However, because the present study is based solely on behavioral data, interpretations regarding neural resource allocation or compensatory mechanisms should be considered speculative and require validation through future neuroimaging research. When compensatory capacity approaches its upper limit, behavioral differences may become more apparent (35). Therefore, high-load task conditions may be more sensitive in revealing working memory differences between T2DM individuals with varying cognitive status.

Overall, these findings further indicate that under conditions of elevated cognitive load, differences in working memory performance between T2DM individuals with varying cognitive status become more apparent, reflecting a load-dependent pattern of impairment.

### 4.2 Relationships between verbal and visuospatial 2-Back accuracy and MMSE performance

The results of the present study showed that accuracy on both the verbal and visuospatial 2-back tasks was significantly positively correlated with MMSE scores (r = 0.461, *p* < 0.01; r = 0.659, *p* < 0.001). Further regression analyses indicated that, after adjusting for relevant covariates, inclusion of accuracy on the verbal and visuospatial 2-back tasks significantly increased the proportion of variance in MMSE scores explained by the model, with corresponding ΔR^2^ values of 0.189 and 0.386, respectively.

This finding is consistent with the pattern reported by Castillo et al. (36) across different working memory tasks. In contrast to the traditional Digit Span (DS) and Spatial Span (SS) tasks used in that study, the present study employed the N-back paradigm to assess working memory. By emphasizing continuous updating and dynamic processing of information, the N-back task more closely reflects everyday working memory demands, thereby facilitating the investigation of the behavioral association between working memory task performance and MMSE scores.

From the perspective of interference theory, forgetting primarily arises from interference among memory traces rather than simple temporal decay (37). Within this framework, spatial information—due to its relatively high perceptual similarity—is more susceptible to interference than verbal information, which typically contains more distinctive cues. Consequently, the encoding and maintenance of spatial information may impose greater cognitive demands. In addition, verbal working memory benefits from subvocal rehearsal mechanisms, whereas visuospatial working memory relies more heavily on selective attention to process, store, and manipulate relevant information while filtering out irrelevant input (21). These characteristics may provide a theoretical explanation for the association observed in the present study between visuospatial 2-back task accuracy and MMSE scores; however, further research is needed to confirm this interpretation.

In summary, in the present sample, accuracy on both verbal and visuospatial 2-back tasks was significantly associated with MMSE scores in cross-sectional analyses. Given the cross-sectional design of this study, the causal direction or predictive relationship between these variables cannot be determined.

### 4.3 Advantages and limitations

The strengths of the present study are primarily reflected in its research design and measurement approach. The use of the N-back task, with automated recording of reaction time and accuracy, enhanced the objectivity of working memory assessment. By distinguishing between verbal and visuospatial working memory tasks and incorporating different levels of cognitive load, this study enabled a more refined characterization of domain-specific working memory performance in individuals with T2DM. Furthermore, the operational grouping of T2DM participants based on MMSE scores facilitated comparisons of working memory characteristics among individuals with different MMSE-defined cognitive statuses within the same disease context.

Several limitations of this study should be acknowledged. First, the cross-sectional design precludes causal inferences regarding the relationships among variables. Second, the verbal and visuospatial N-back tasks, as well as the different cognitive load conditions, were administered in a fixed order, which may have introduced potential order or fatigue effects. Third, the inclusion of only hospitalized patients may limit the generalizability of the findings. In addition, global cognitive function was primarily assessed using the MMSE, which has limited sensitivity for detecting subtle or early cognitive changes. The grouping strategy employed in this study was intended solely for research purposes and does not constitute a formal clinical diagnosis of cognitive impairment. Moreover, the diagnostic or screening utility of the N-back task for cognitive impairment was not evaluated; thus, the present findings reflect statistical associations only. Furthermore, depressive and anxiety symptoms were not quantitatively assessed using standardized scales, which may have resulted in residual confounding and increased uncertainty in interpreting differences in N-back task performance. Finally, the absence of biomarker and neuroimaging data limited further exploration of the underlying pathophysiological mechanisms.

Future studies should consider adopting longitudinal designs, extending recruitment to community-based populations, and incorporating more sensitive cognitive assessment tools along with multimodal biomarkers. Such approaches would help to further validate the robustness of the present findings and provide deeper insight into the underlying mechanisms.

## 5 Conclusion

Overall, at the cross-sectional level, verbal and visuospatial working memory performance in patients with T2DM was significantly associated with global cognitive status, with between-group differences most pronounced under high cognitive load conditions. In the present sample, visuospatial 2-back performance showed a numerically stronger association with global cognitive status. This finding is exploratory in nature and warrants further validation in future studies.

## Data Availability

The original contributions presented in the study are included in the article/Supplementary material, further inquiries can be directed to the corresponding authors.
