# Supplementary material for: Working memory characteristics in T2DM individuals with MMSE-defined cognitive status: a behavioral study
Source: Front Neurol. 2026 Mar 20;17:1751377. doi: 10.3389/fneur.2026.1751377 (PMC13046544; doi:10.3389/fneur.2026.1751377)
Supplement: Supplementary file 1 [file Table_1.docx]

Supplementary Material

**Supplementary Table.**

**Table S1. Sensitivity analysis of reaction time using repeated-measures ANOVA**

|  |  | SS | df | MS | *F* | *P* | η² |
| --- | --- | --- | --- | --- | --- | --- | --- |
| Verbal N-back reaction time (ms) | Group^a^ | 1042476.333 | 1 | 1042476.333 | 58.328 | ＜0.001 | 0.543 |
|  | Load level^b^ | 10152355.74 | 2 | 5076177.870 | 400.702 | ＜0.001 | 0.891 |
|  | Group*Load level^c^ | 104958.390 | 2 | 52479.195 | 4.143 | 0.019 | 0.078 |
| Verbal N-back accuracy(%) | Group^a^ | 305.750 | 1 | 305.750 | 9.199 | 0.004 | 0.158 |
|  | Load level^b^ | 22547.969 | 2 | 11273.985 | 635.045 | ＜0.001 | 0.928 |
|  | Group*Load level^c^ | 317.396 | 2 | 258.698 | 12.297 | ＜0.001 | 0.191 |
| Visuospatial N-back reaction time(ms) | Group^a^ | 1322204.089 | 1 | 1322204.089 | 58.614 | ＜0.001 | 0.545 |
|  | Load level^b^ | 10712110.79 | 2 | 5356055.397 | 404.663 | ＜0.001 | 0.892 |
|  | Group*Load level^c^ | 128426.314 | 2 | 64213.157 | 4.851 | 0.010 | 0.090 |
| Visuospatial N-back Accuracy(%) | Group^a^ | 602.206 | 1 | 602.206 | 15.738 | ＜0.001 | 0.232 |
|  | Load level^b^ | 29638.489 | 2 | 14819.245 | 971.984 | ＜0.001 | 0.952 |
|  | Group*Load level^c^ | 262.466 | 2 | 131.233 | 8.607 | ＜0.001 | 0.149 |

a represents the main effect of group, b represents the main effect of load level, and c represents the interaction effect between group and load level; SS, sum of squares; df, degrees of freedom; MS, mean square；η²，eta-squared.

**Table S2. Sensitivity analysis of reaction time using partial correlation analysis**

|  | *r* | *P* |
| --- | --- | --- |
| Verbal N-back task accuracy(%) | | |
| 0-back | 0.099 | 0.502 |
| 1-back | 0.208 | 0.155 |
| 2-back | 0.445 | 0.002 |
| Visuospatial N-back task accuracy(%) | | |
| 0-back | 0.107 | 0.471 |
| 1-back | 0.186 | 0.206 |
| 2-back | 0.644 | ＜0.001 |

**Table S3. Sensitivity analysis of reaction time using hierarchical regression analysis**

| variable | Verbal Task | | Visuospatial Task | |
| --- | --- | --- | --- | --- |
|  | Model 1 | Model 2 | Model 1 | Model 2 |
| Constant | 1.532** | 1.461** | 1.726** | 1.744** |
| Age | -0.044 | -0.123 | -0.044 | -0.115 |
| Sex | -0.734 | -0.783 | -0.734 | -1.386 |
| Education | 0.697 | 0.419 | 0.697 | 0.055 |
| 2-back Accuracy | / | 0.248** | / | 0.315*** |
| R^2^ | 0.115 | 0.290** | 0.115 | 0.482*** |
| △R^2^ | 0.058 | 0.228** | 0.058 | 0.437*** |
| F | 2.026 | 4.697** | 2.026 | 10.712*** |

(1)The table shows unstandardized regression coefficients (B).(2)Model 1 includes age, sex, and years of education as control variables. Model 2 further includes 2-back task accuracy.(3)The incremental contribution of working memory performance to the model is assessed through the change in explained variance (ΔR²), rather than directly comparing the size of the regression coefficients.(4)p < 0.05, *p < 0.01, **p < 0.001.(5)"Accuracy" in the table refers to the behavioral performance metric of the 2-back task and is not used as a clinical diagnostic criterion.

**Table S4. Sensitivity analyses examining the association between 2-back accuracy and reaction time after controlling for disease severity**

| Disease severity indicator | Model 1 | Model 2 | △R^2^ |
| --- | --- | --- | --- |
| Verbal task | | | |
| Diabetes duration (years) | B=6.355,P=0.122 | B=-13.078,P=0.003 | 0.159 |
| HbA1c (%) | B=6.815,P=0.478 | B=-13.902,P=0.002 | 0.173 |
| Number of comorbidities | B=41.930,P=0.063 | B=-13.153,P=0.002 | 0.163 |
| Visuospatial task | | | |
| Diabetes duration (years) | B=4.885,P=0.227 | B=-17.464,P＜0.001 | 0.281 |
| HbA1c (%) | B=7.295,P=0.438 | B=-18.129,P＜0.001 | 0.291 |
| Number of comorbidities | B=36.941,P=0.096 | B=-17.319,P＜0.001 | 0.280 |

Model 1 controlled for age, sex, and years of education and included one disease severity indicator at a time. Model 2 additionally included 2-back accuracy. The coefficients reported in Model 1 correspond to the disease severity indicator, whereas those reported in Model 2 correspond to 2-back accuracy. ΔR² represents the increase in explained variance after adding 2-back accuracy.

**Table S5. Sensitivity analyses of the association between 2-back accuracy and MMSE after adjustment for disease severity indicators**

| Disease severity indicator | B（2-back accuracy） | P | R^2^ |
| --- | --- | --- | --- |
| Verbal task | | | |
| None (main model) | 0.260 | 0.002 | 0.301 |
| Diabetes duration (years) | 0.244 | 0.002 | 0.291 |
| HbA1c (%) | 0.222 | 0.003 | 0.362 |
| Number of comorbidities | 0.228 | 0.003 | 0.335 |
| Visuospatial task | | | |
| None (main model) | 0.316 | ＜0.001 | 0.498 |
| Diabetes duration (years) | 0.313 | ＜0.001 | 0.483 |
| HbA1c (%) | 0.290 | ＜0.001 | 0.518 |
| Number of comorbidities | 0.303 | ＜0.001 | 0.530 |

B represents the unstandardized regression coefficient for 2-back accuracy, P indicates the corresponding level of statistical significance, and R² denotes the proportion of variance in MMSE explained by each model. “None (main model)” refers to the primary model without adjustment for any disease severity indicators and serves as the reference.
